# Interconnections between apoptotic and autophagic pathways during thiopurine-induced toxicity in cancer cells: the role of reactive oxygen species

**DOI:** 10.18632/oncotarget.12313

**Published:** 2016-09-28

**Authors:** Wiem Chaabane, Malin Lindqvist Appell

**Affiliations:** ^1^ Division of Drug Research, Department of Medical and Health Sciences, Linköping University, SE-58183 Linköping, Sweden

**Keywords:** 6-thioguanine, 6-mercaptopurine, azathioprine, autophagy, apoptosis

## Abstract

Thiopurines (azathioprine, 6-mercaptopurine and 6-thioguanine) are a class of genotoxic drugs extensively used in the treatment of various illnesses including leukemia. Their underlying molecular mechanism of action involves the activation of apoptosis and autophagy but remains widely unclear. Here we present evidence that autophagy induction by thiopurines is a survival mechanism that antagonizes apoptosis and is involved in degrading damaged mitochondria through mitophagy. On the other hand, apoptosis is the main cell death mechanism by thiopurines as its inhibition prohibited cell death. Thus a tight interplay between apoptosis and autophagy controls cell fate in response to thiopurine treatment. Moreover, thiopurines disrupt mitochondrial function and induce a loss of the mitochondrial transmembrane potential. The involvement of the mitochondrial pathway in thiopurine-induced apoptosis was further confirmed by increased formation of reactive oxygen species (ROS). Inhibiting oxidative stress protected the cells from thiopurine-induced cell death and ROS scavenging prohibited autophagy induction by thiopurines. Our data indicate that the anticarcinogenic effects of thiopurines are mediated by complex interplay between cellular mechanisms governing redox homeostasis, apoptosis and autophagy.

## INTRODUCTION

The thiopurines azathioprine (AZA), 6-mercaptopurine (6-MP) and 6-thioguanine (6-TG) are chemotherapeutic agents used in treatment of different disorders [1, 2]. 6-MP and 6-TG are mainly used in the treatment of leukemia while AZA is extensively used in the treatment of inflammatory bowel disease (IBD, including ulcerative colitis and Crohn's disease) [3, 4]. Before exerting their action, thiopurines undergo extensive metabolism. AZA, a prodrug of 6-MP, is activated to 6-MP through a conjugation reaction with glutathione (GSH), which can lead to the depletion of GSH. This conversion can occur spontaneously or by enzymatic conversion through glutathione S-transferase, the later leading to reactive oxygen species (ROS) [5]. 6-MP is metabolized by xanthine oxidase (XO) to thiouric acid, a reaction that is also known to create ROS [6]. Two other alternative routes decides the fate of 6-MP; 6-MP can be inactivated by thiopurine methyltransferase (TPMT) or converted to cytotoxic 6-TG metabolites by hypoxanthine-guanine phosphoribosyltransferase (HPRT) and subsequent reactions. 6-TG is metabolized to 6-thioguanosine monophosphate by hypoxanthine-guanine phosphoribosyl transferase (HPRT), then phosphorylated to 6-thioguanosine triphosphate through a series of enzymatic reactions and is then incorporated into genomic DNA [7]. In DNA, the incorporated 6-TG is further methylated by S-adenosylmethionine to form S6-methylthioguanine [8]. S6-methylthioguanine pairs with thymine and its normal partner, cytosine, during subsequent DNA replication. 6-TG induced mispairs are processed by the DNA mismatch repair (MMR), which leads to an initial prolonged cell cycle arrest followed by apoptosis and autophagy [7, 9]. Although widely used, many gaps persist in the knowledge of how these drugs exert their action as to the pathways involved in their pharmacological and toxicological effects. This lack of knowledge is exemplified by the fact that 10 to 45% of IBD patients do not respond adequately to thiopurines. One of the dark spots in thiopurine mechanism is the role played by cell autophagy and its interplay with apoptosis.

Autophagy is a regulated cell survival process of degradation and recycling of cellular constituents within a unique organelle called the autophagosome, during organelle turnover or starvation management [10, 11]. Nevertheless, beyond its basal level, autophagy is a type of cell demise, activated upon excessive cellular damage and defined as type II cell death as opposed to the type I non-autophagic, apoptotic cell death [12]. Apoptotic cell death or apoptosis is the prevalent form of programmed cell death (PCD) in multicellular organisms induced by various stimuli including chemotherapy and DNA damage. It is characterized, morphologically, by cell shrinkage, chromatin condensation and formation of apoptotic bodies and biochemically, by caspase activation and DNA fragmentation [13]. Two partly interconnected apoptotic mechanisms exist, caspase-dependent and caspase-independent apoptosis, sometimes called necroptosis [14, 15]. Both forms of cell death may be interconnected as caspases may lead to the activation of non-caspase proteases and vice versa. The classical, caspase-dependent apoptosis induce apoptosis within two distinct pathways: the extrinsic or the intrinsic pathways. Both converge on the same execution pathway, which is initiated by the cleavage of caspase-3 [16]. The intrinsic apoptotic pathway can be divided into three major phases: a pre-mitochondrial phase during which signal transduction cascades are activated; a mitochondrial phase, during which a loss of the mitochondrial membrane potential occurs; and a post-mitochondrial phase, during which the release of mitochondrial proteins lead to the activation of catabolic proteases and nucleases including caspases [13]. Alterations in mitochondria transmembrane potential (ΔΨm) lead to the production of reactive oxygen species (ROS) or mitochondrial membrane permeabilization [17].

Since autophagy is activated upon cellular stress, which can also lead to apoptosis, various interactions between these two forms of cell death have been defined; 1) Autophagy can function as a survival mechanism that antagonizes apoptosis via limiting ER stress induced by environmental factors and maintaining its normal function [18, 19]. 2) Autophagy can also function as a facilitator of apoptosis via maintaining ATP levels required for ATP-dependent apoptotic processes, including proper apoptotic bodies formation and removal [19]. 3) Autophagy can cooperate with apoptosis by either functioning in parallel or in sequence to the apoptotic process and regulates caspase activity [20–22]. 4) Autophagy can also be a selective mechanism involved in the turnover of damaged organelles such as mitochondria (mitophagy) [23–25]. In this context, damaged mitochondria can activate concomitantly apoptosis and mitophagy and the balance between the two determines whether a cell will live or die. Enhanced mitophagy promote survival by removing damaged mitochondria. When mitochondria damage exceeds the threshold, apoptosis becomes dominant, and inactivation of critical proteins of the autophagy pathway allows for cell death to occur.

A previous report indicated that both PCD pathways I and II are induced within the same cells after MMR processing of 6-TG [26]. However, the molecular mechanisms of this MMR-mediated drug cytotoxicity as to the role of autophagy activation and its interplay with apoptosis are not yet precisely elucidated.

In this study, we investigated the molecular mechanism of thiopurine toxicity in MMR-proficient colorectal cancer cell lines. We found that autophagy activation by all three thiopurines 6-MP, AZA and 6-TG is a cell defense mechanism that counteracts apoptosis. In addition, we show that apoptosis is the main cell death form by all three thiopurines as inhibiting apoptosis significantly hampered cell death. Moreover, thiopurines induce mitochondrial damage and loss of the mitochondrial transmembrane potential, which is even greater when autophagy is inhibited. Autophagy activation by 6-TG is a selective process involved in eliminating damaged mitochondria through mitophagy as both mitochondria and autophagy signal colocalizes. Furthermore, we show that ROS play an important role in thiopurine-triggered cell death and may serve as a messenger between the mitochondrial and the lysosomal pathways. Together, the data generated in this manuscript give an insight to the molecular machinery involved in the cytotoxicity of thiopurines.

## RESULTS

### Suppression of autophagy by chloroquine sensitizes 6-TG mediated cell death in colorectal cancer cell lines

Autophagy induces the sequestration of cytoplasmic organelles into double-layered vesicles, called autophagosomes. Autophagosomes fuse with endosomes and thereafter with lysosomes, thus creating autophago-lysosomes or autolysosomes. In the lumen of these latter structures, lysosomal enzymes catabolize the autophagic material [24].

**Figure 1:**
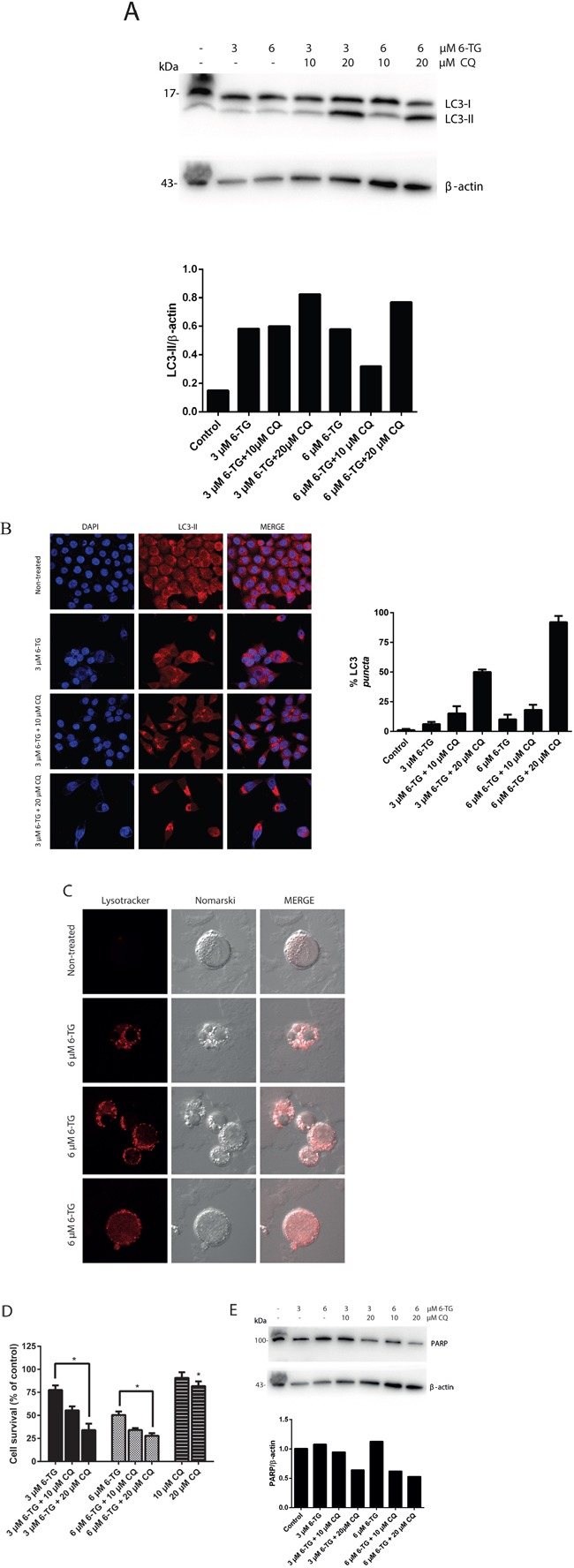
Suppression of autophagy by chloroquine sensitizes 6-TG mediated cell death in colorectal cancer cell lines **A.** Analysis of LC3 protein level after 6-TG treatment of HCT116 MMR-proficient colorectal cancer cells. Cells were treated with 6-TG (3 μM or 6 μM) for 24 hours followed by 6 hours treatment with CQ (10 μM or 20 μM) and analyzed by western blotting 72 hours later. Histogram shows the quantitation of LC3-II relatively to β-actin. **B.** Visualization of LC3 by confocal microscopy. HCT116 cells were exposed to 6-TG (6 μM) for 24 hours and stained with an antibody to LC3 72 hours post treatment. The control cells showed diffuse cytosolic LC3 distribution. 6-TG treated cells showed an increase in the number of cells with LC3 dots, which is even higher upon CQ coexposure. Histogram shows the percentage of cells with LC3 puncta. Results are the mean of three experiments ± SEM. **C.** Visualization of lysosomes by confocal microscopy HCT116 MMR-proficient cells were exposed to 6-TG (6 μM) for 24 hours and stained with the acidophilic lysosomal probe lysotracker red (LTR) 72 hours post treatment. 6-TG caused an increase in the volume and frequency of cytoplasmic granules staining with LTR. 6-TG-induced autophagy involved lysosomal activation. **D.** Analysis of cell survival in HCT116 MMR-proficient cells. Cells were exposed to 6-TG for 24 hours and viability was determined 72 hours later using po-pro/7AAD staining and flow cytometry. Results are expressed as percentage cell survival and represent the mean ± SEM of three independent experiments. CQ cotreatment significantly increased apoptosis induction by 6-TG (*P* ≤ 0.05). **E.** Western blot analysis of PARP level of expression in HCT116 MMR-proficient cells treated as in A. Histogram shows the quantitation of PARP relatively to β-actin. Autophagy inhibition using CQ increased PARP cleavage and sensitized the cells to cell death by 6-TG.

To investigate autophagy, HCT116 MMR-proficient cells were used. In these cells, MLH1 activity has been restored through stable transfection with the human MLH1 DNA, (Supplementary Figure S1) [27]. HCT116 MMR-proficient cells were treated with different concentrations of 6-TG (3 μM or 6 μM) for 24 hours then either left untreated or were cotreated for 6h with chloroquine (CQ, 10 μM or 20 μM), a late phase autophagy inhibitor, which prevents the autophagosomal degradation. The expression pattern of LC3-I and LC3-II were then investigated using the corresponding specific antibody as described in the material and methods section. The levels of LC3-II protein were increased upon exposure to 6-TG in HCT116 MMR-proficient cells (Figure 1A) and in HT29 cells (Supplementary Figure S2A). The levels of LC3-II were further increased upon coexposure with CQ consistent with a pronounced autophagic flux in 6-TG exposed cells. These data indicate that 6-TG stimulates the conversion of a significant fraction of LC3-I to LC3-II in MMR-proficient cells. To confirm these data, the intracellular localization of LC3 was visualized by fluorescence microscopy, which is another specific and reliable method to monitor autophagy in mammalian cells [28]. In non-treated cells, prior to induction of autophagy, LC3 shows diffuse localization in the cytosol; whereas when autophagy is induced by 6-TG; LC3 specifically relocalizes to autophagic membranes and exhibits punctate dot cytoplasmic signals. CQ cotreated cells show an increase in the number of cells exhibiting an LC3 dot cytoplasmic signal (Figure 1B). On the other hand, 6-TG treatment of HCT116 MMR-proficient cells caused an increase in both volume and frequency of cytoplasmic granules after staining with lysotracker (Figure 1C). Thus 6-TG induces lysosome activation and autophagy in MMR-proficient cells.

Next, the effect of autophagy suppression by CQ on the cytotoxicity of 6-TG was investigated. CQ coexposure significantly enhanced cell death induction by 6-TG in HCT116 MMR-proficient cells (Figure 1D) and in HT29 cells (Supplementary Figure S2B). The increase in the apoptotic rates after CQ coexposure was further confirmed by the decrease in the level of full length PARP, corresponding to an increase of PARP cleavage (an apoptosis marker). CQ cotreatment enhanced 6-TG-induced PARP cleavage in a dose-dependent manner in HCT116 MMR-proficient cells (Figure 1E) and in HT29 cells (Supplementary Figure S2C). These data indicate that blocking autophagy by CQ enhances 6-TG-induced apoptosis. We conclude that autophagy induction by 6-TG in MMR-proficient cells is a cell defense mechanism that counteracts cell death by 6-TG.

### Suppression of autophagy by chloroquine sensitizes 6-MP and AZA mediated cell death in colorectal cancer cell lines

**Figure 2:**
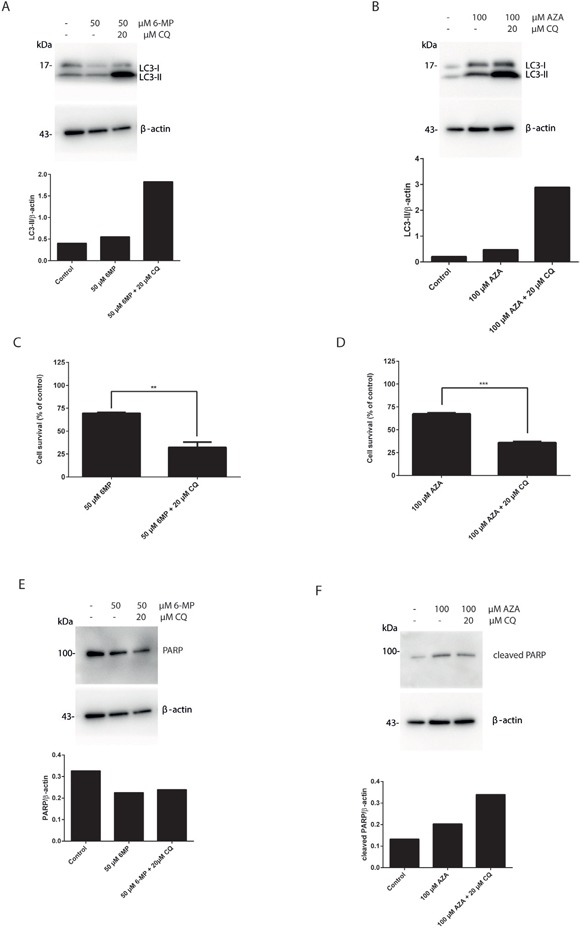
Suppression of autophagy by chloroquine sensitizes 6-MP and AZA mediated cell death in colorectal cancer cell lines **A.** Analysis of LC3 expression in HT29 colorectal cancer cells. Cells were treated with 6-MP (50 μM) for 24 hours followed by 6 hours treatment with CQ (20 μM) and analyzed by western blotting 72 hours post treatment. Histogram shows the quantitation of LC3-II relatively to β-actin. **B.** Analysis of LC3 expression after AZA treatment of HT29 cells. Cells were treated with AZA (100 μM) for 24 hours followed by 6 hours treatment with CQ (20 μM) and analyzed by western blotting 72 hours post treatment. Histogram shows the quantitation of LC3-II relatively to β-actin. **C.** Analysis of cell survival in HT29 cells. Cells were exposed to 50 μM 6-MP for 24 hours followed by 6 hours treatment with 20 μM CQ. Viability was determined after 72 hours using po-pro/7AAD staining and flow cytometry. Results are expressed as percentage of cell survival and represent the mean ± SEM of three independent experiments. CQ cotreatment significantly increased apoptosis induction by 6-MP (*P* ≤ 0.01). **D.** Flow cytometry analysis of cell survival of HT29 cells treated with AZA and cotreated with CQ. HT29 cells were exposed to 100 μM AZA for 24 hours followed by 6 hours treatment with 20 μM CQ. Viability was determined 72 hours later using po-pro/7AAD staining and flow cytometry. Results are expressed as percentage of cell survival and represent the mean ± SEM of three independent experiments. CQ cotreatment significantly increased apoptosis induction by AZA (*P* ≤ 0.001). **E.** Western blot analysis of PARP level of expression in HT29 cells treated as in A. Histogram shows the quantitation of PARP relatively to β-actin. Autophagy inhibition using CQ decreased PARP level and sensitized the cells to cell death by 6-MP. **F.** Western blot analysis of PARP cleavage in HT29 cells treated as in B. Histogram shows the quantitation of cleaved PARP relatively to β-actin. Autophagy inhibition using CQ increased PARP cleavage level and sensitized the cells to cell death by AZA.

In order to give more insight into the molecular machinery governing thiopurine cytotoxicity, the possible activation of autophagy by AZA and 6-MP, the more commonly used thiopurines clinically, was investigated in HT29 cells. HT29 cells were treated either with 6-MP (30 μM or 50 μM) or AZA (50 μM or 100 μM) for 24 hours. LC3 conversion (LC3-I to LC3-II) was determined in the presence or in the absence of CQ coexposure. The levels of LC3-II protein were increased after coexposure of either 6-MP and CQ (Figure 2A), or AZA and CQ (Figure 2B). Thus, both AZA and 6-MP activate autophagy in HT29 cells. Moreover, inhibiting autophagy using CQ significantly increased cell death in response to 6-MP (Figure 2C) or AZA (Figure 2D). The level of PARP was decreased in response to 6-MP and CQ cotreatment (Figure 2E) and the level of PARP cleavage was increased in response to AZA and CQ cotreatment (Figure 2F), consistent with an increase in apoptotic rates. Thus similarly to 6-TG, autophagy activation by 6-MP and AZA is also a cell protective mechanism that counteracts cell death.

### Apoptosis is the main pathway of cytotoxicity of thiopurines and is caspase dependent

**Figure 3:**
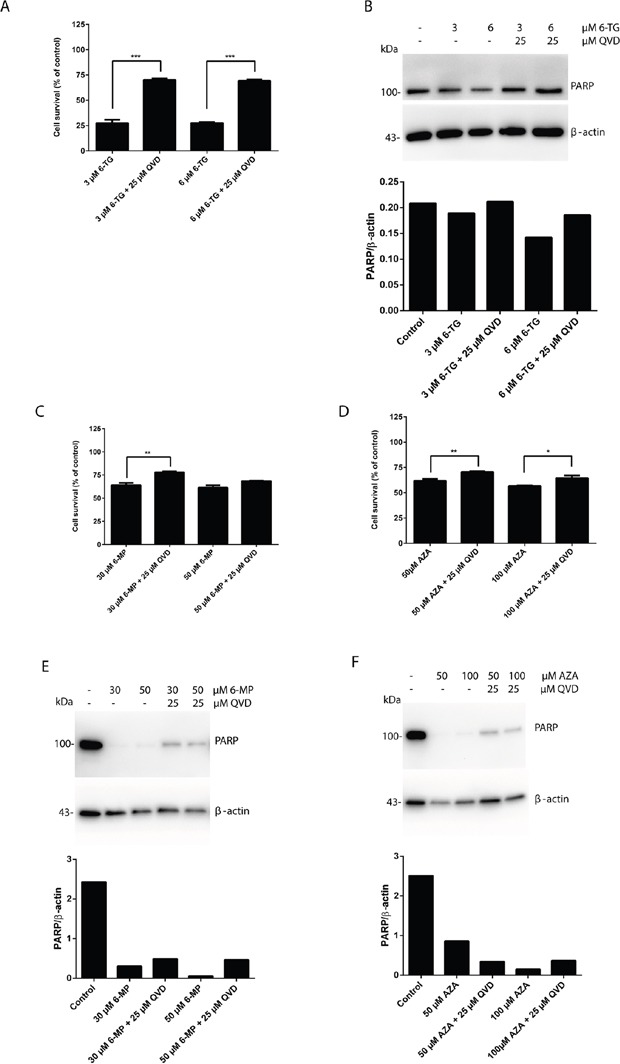
Apoptosis is the main pathway of cytotoxicity of thiopurines, which is caspase dependent **A.** Quantitation of cell survival in HCT 116 MMR-proficient cells. Cells were allowed to attach for 10 hours prior to 6-TG (3 μM or 6 μM) treatment. Cells were cotreated with QVD (25 μM) for caspase inhibition and were analyzed by flow cytometry 72 hours post treatment. Results are expressed as percentage of cell survival and represent the mean ± SEM of three independent experiments. QVD cotreatment significantly protected the cells from cell death in response to 6-TG (*P ≤* 0.001). **B.** Western blot analysis of PARP level of expression in response to caspase inhibition by QVD in cells treated as in A. Histogram shows the quantitation of PARP relatively to β-actin. **C.** Analysis of cell survival in cells treated with 6-MP (30 μM or 50 μM) and cotreated with QVD (25 μM) for caspase inhibition by flow cytometry 72 hours post treatment. Results are expressed as percentage of cell survival and represent the mean ± SEM of three independent experiments. QVD cotreatment significantly protected the cells from apoptosis induction by 6-MP (*P* ≤ 0.01). **D.** Analysis of cell survival in cells treated with AZA (50 μM or 100 μM) and cotreated with QVD (25 μM) for caspase inhibition by flow cytometry 72 hours post treatment. Results are expressed as percentage of cell survival and represent the mean ± SEM of three independent experiments. QVD cotreatment significantly protected the cells from apoptosis induction by AZA (*P* ≤ 0.01). **E.** Western blot analysis of PARP level of expression in response to caspase inhibition by QVD in cells treated as in C. Histogram shows the quantitation of PARP relatively to β-actin. Caspase inhibition decreases PARP cleavage in response to 6-MP treatment. **F.** Western blot analysis of PARP level of expression in response to caspase inhibition by QVD in cells treated as in D. Histogram shows the quantitation of PARP relatively to β-actin. Caspase inhibition decreases PARP cleavage in response to AZA treatment.

Certain forms of apoptosis could be efficiently counteracted by the inhibition of caspases. Therefore, we analyzed the effect of caspase inhibition on the cytotoxicity of 6-TG using a broad range caspase inhibitor (QVD). The broad-spectrum caspase inhibition significantly hampered 6-TG induced cell death (Figure 3A). In addition, the level of PARP was increased when the cells were cotreated with QVD (Figure 3B). Thus, 6-TG activates ultimately the apoptotic cell death pathway. Apoptosis occur in a caspase dependent manner. Moreover, inhibiting caspases by QVD also diminished the cytotoxicity of 6-MP and AZA further supporting that apoptosis is the major cell death pathway involved in the cytotoxicity of all three thiopurines as shown by the cell survival assay (Figure 3A, 3C and 3D) and the level of full length PARP (Figure 3B, 3E and 3F).

### Autophagy induction by thiopurines is solely a cell protective mechanism

**Figure 4:**
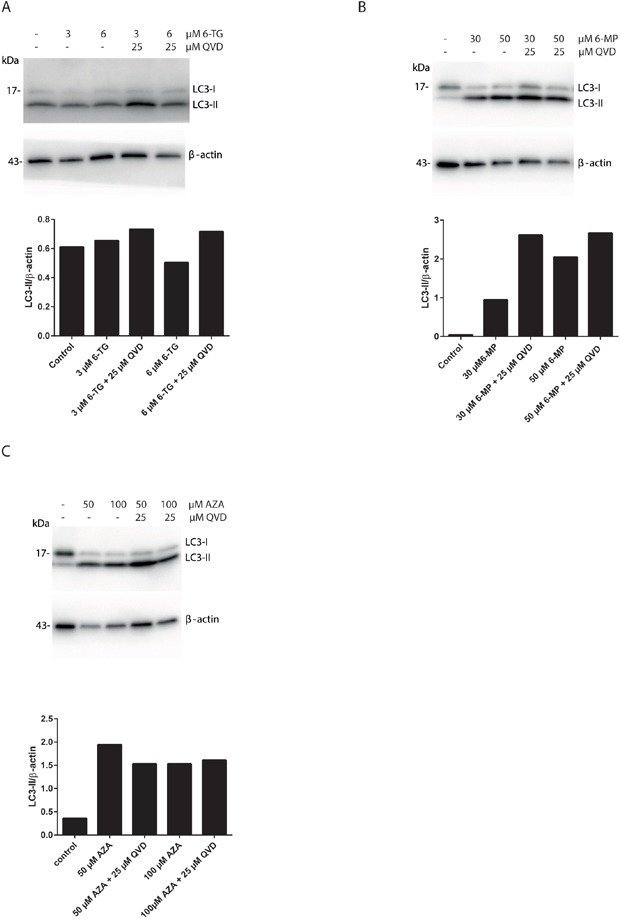
Apoptosis inhibition using QVD promotes autophagy induction in thiopurines treated cells **A.** Analysis of LC3 expression in HCT116 MMR-proficient cells. Cells were treated with 6-TG (3 μM or 6 μM) and cotreated with QVD (25 μM) for caspase inhibition. LC3 level of expression were determined by western blotting 72 hours post treatment. Histogram shows the quantitation of LC3-II relatively to β-actin. **B.** Analysis of LC3 expression in HCT116-MMR proficient cells by western blotting 72 hours post treatment with 6-MP (30 μM or 50 μM) and cotreatment with QVD (25 μM). Histogram shows the quantitation of LC3-II relatively to β-actin. **C.** Analysis of LC3 expression in HCT116-MMR expressing cells by western blotting 72 hours post treatment with AZA (50 μM or 100 μM) and cotreatment with QVD (25 μM) for caspase inhibition. Histogram shows the quantitation of LC3-II level relatively to β-actin.

The removal or functional inhibition of essential proteins from the apoptotic machinery can de-inhibit autophagy or switch a cellular stress response from the apoptotic default pathway to a state of massively increased autophagy and autophagic cell death [29]. To further check the interplay between autophagy and apoptosis signaling during MMR processing of thiopurines, the effect of apoptosis inhibition on autophagy was investigated. Cells exposed to 6-TG, when co-treated with QVD, showed a greater autophagic response suggesting that apoptosis inhibits autophagy during 6-TG-induced cytotoxicity (Figure 4A). Moreover, the same effect was observed in 6-MP (Figure 4B) and to a lesser extent in AZA exposed cells (Figure 4C), when co-treated with QVD. Thus inhibiting apoptosis while stimulating autophagy does not switch autophagy from a cell protective to a cell death pathway.

### Thiopurine-induced cell death is at least in part due to the intrinsic apoptotic death pathway

**Figure 5:**
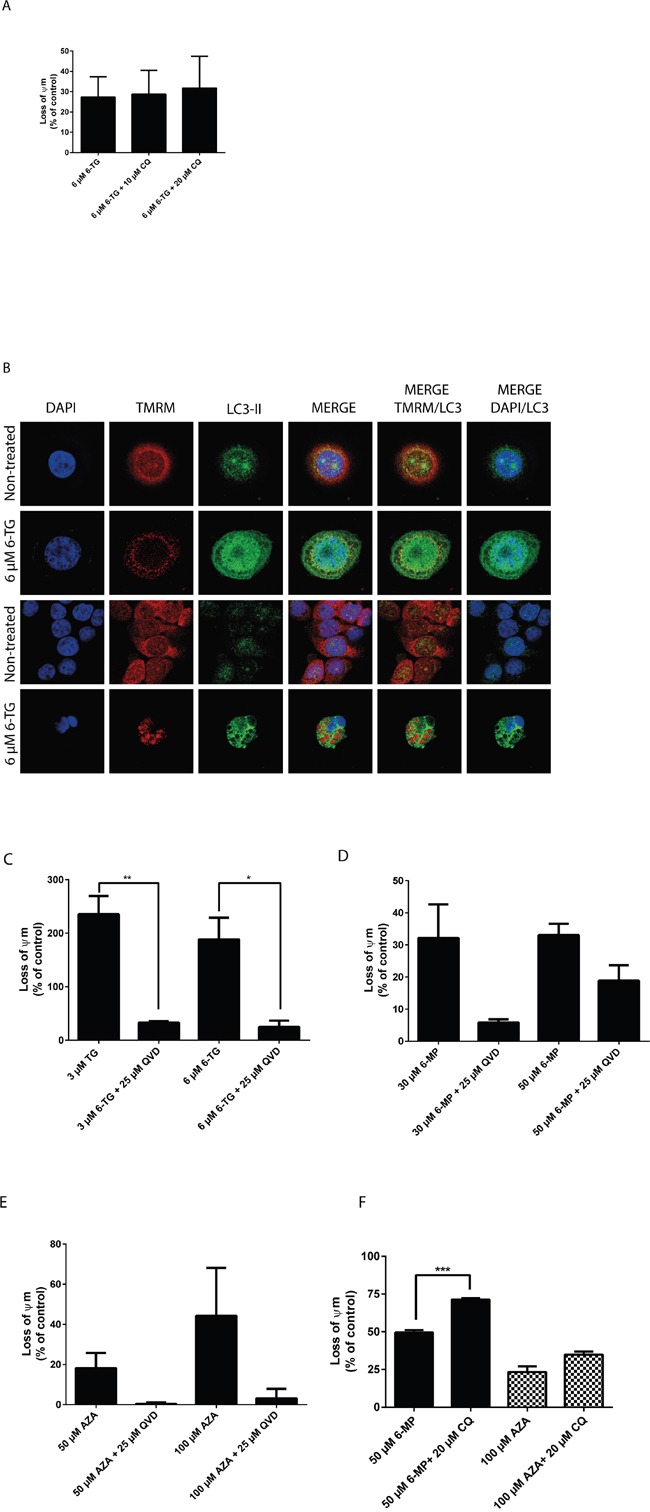
Thiopurines induced cell death is at least in part due to the intrinsic apoptotic death pathway **A.** Measurement of the mitochondrial potential (Ψm) in HCT116 MMR-expressing cells treated with 6-TG (3 μM or 6 μM) and cotreated with CQ (10 μM or 20 μM). Ψm was determined using the TMRM fluorescent dye and flowcytometry. 6-TG treatment induces a marked decrease in Ψm, which is even greater upon CQ cotreatment. **B.** Co-localization of the autophagic marker LC3-II and the mitochondria dye TMRM in HCT116-MMR proficient cells treated with 6-TG. Cells were exposed to 6-TG (6 μM) for 24 hours. 72 hours later, cells were stained with TMRM for 15 minutes and then counterstained with LC3-II and analyzed by confocal microscopy. The TMRM dye is rapidly sequestrated by healthy mitochondria while the depolarized mitochondria do not accumulate the dye. The non-treated cells show preserved mitochondria whereas cells treated with 6-TG show disruption in the mitochondrial network and a weak mitochondrial signal relative to damaged mitochondria. Moreover, 6-TG treated cells show LC3-II-associated autophagosomal puncta formation as compared to control cells showing diffuse cytosolic LC3 distribution. Mitochondrial autophagy was further confirmed by LC3 signal colocalization with the mitochondria signal (TMRM). **C.** Measurement of Ψm in HCT116 MMR-proficient cells treated with 6-TG (3 μM or 6 μM) and cotreated with QVD (25 μM). Results are expressed as percentage loss of Ψm and represent the mean ± SEM of three independent experiments. QVD cotreatment significantly protected cells (*P* ≤ 0.01) from the loss of Ψm when exposed to 6-TG. **D.** Measurement of Ψm in HCT116 MMR-proficient cells treated with 6-MP. Results are expressed as percentage loss of Ψm and represent the mean ± SEM of three independent experiments. 6-MP treatment (30 μM or 50 μM) induces a significant decrease in Ψm (*P* ≤ 0.001). QVD cotreatment protected the cells from the loss of Ψm when exposed to 6-MP. **E.** Measurement of Ψm in HCT116-MMR-expressing cells treated with AZA. Results are expressed as percentage loss of Ψm and represent the mean ± SEM of three independent experiments. AZA treatment (50 μM or 100 μM) induces a decrease in Ψm. QVD cotreatment (25 μM) protected the cells from the loss of Ψm when exposed to AZA. **F.** Measurement of Ψm in HT29 cells treated with 6-MP (50 μM) or AZA (100 μM) and cotreated with CQ (20 μM). Results are expressed as percentage loss of Ψm and represent the mean ± SEM of three independent experiments. CQ cotreatment (25 μM) enhanced the loss of Ψm in cells when exposed to 6-MP or AZA.

In order to evaluate mitochondrial changes induced by thiopurines, the tetramethylrhodamine methyl ester (TMRM) dye was used. TMRM is rapidly sequestrated by healthy mitochondria while the depolarized mitochondria do not accumulate the dye [30]. Cells treated with 6-TG showed a pronounced decrease in TMRM fluorescence compared to the non-treated control cells consistent with a loss of the mitochondrial transmembrane potential. Moreover, CQ cotreatment renders the loss of the mitochondrial potential even more pronounced (Figure 5A, supplementary Fig S3).

Next, the colocalization of the mitochondrial marker TMRM and the autophagic marker LC3-II were analyzed in HCT116 MMR-proficient cells after treatment with 6 μM 6-TG for 24 hours and staining with TMRM (Figure 5B). 6-TG treated cells show low mitochondrial signal due to depolarized mitochondria that fail to retain the dye, as compared to the respective control cells that show strong mitochondrial signal relative to intact mitochondria. Moreover, LC3-II levels were significantly increased in 6-TG treated cells: LC3-II fluorescence displayed a punctuated pattern that is a characteristic feature of autophagy. LC3-II signal also colocalized with the mitochondria signal in these cells, indicating the fusion of mitochondria with autophagic vacuoles. Thus, autophagy activation is involved in degrading damaged mitochondria through mitophagy.

On the other hand, QVD cotreatment significantly protected the cells from the loss of the mitochondrial potential when exposed to 6-TG, further supporting the role of caspases in apoptosis induction by 6-TG (Figure 5C).

These data demonstrate that the mitochondrial pathway plays an important role in 6-TG induced cell death. 6-TG induce mitochondrial damage and loss of the mitochondrial membrane potential concomitantly to the activation of autophagy (mitophagy) and the degradation of damaged mitochondria.

Moreover, HCT116 MMR-proficient cells showed a decrease in the mitochondrial potential when treated with 6-MP or AZA. The mitochondrial potential was preserved when cells were cotreated with QVD (Figure 5D and 5E), while the loss of the mitochondrial potential was enhanced upon cotreatment with CQ (Figure 5F), suggesting a mechanism of action that is common to all three thiopurines.

### Thiopurine treatment leads to the induction of reactive oxygen species

Having shown that 6-TG treatment led to the concomitant induction of autophagy and apoptosis, we opted to elucidate the mechanism governing the interplay between apoptosis and autophagy. In combination with loss of the mitochondrial membrane potential, an increased production of reactive oxygen species (ROS) is often linked to autophagy and apoptosis.

**Figure 6:**
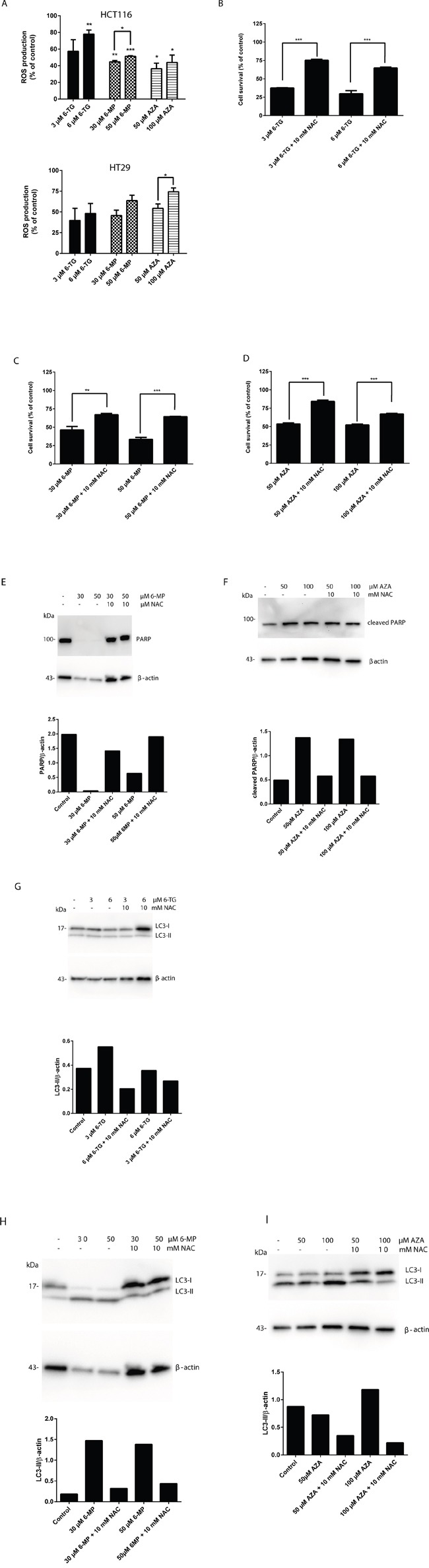
Thiopurines treatment leads to the induction of reactive oxygen species A. Measurement of ROS production in HCT116 MMR-proficient cells and HT29 cells 72 hours post 24 hours treatment with 6-TG (3 μM or 6 μM) or 6-MP (30 μM or 50 μM) or AZA (50μM or 100μM). 6-TG, 6-MP or AZA triggered significantly ROS production in HCT116 cells (*P* ≤ 0.01, P≤0.001 and P≤0.001 respectively) and in HT29 cells (*P* ≤ 0.001, P≤0.05 and P≤0.05 respectively). **B.** Quantitation of cell survival using flow cytometry in HCT116 MMR-proficient cells treated with 6-TG (3 μM or 6 μM) and pretreated with the ROS inhibitor NAC (10mM). Results are expressed as percentage of cell viability and represent the mean ± SEM of three independent experiments. NAC cotreatment significantly hampered apoptosis induction by 6-TG (*P* ≤ 0.001). **C.** Quantitation of cell survival using flow cytometry in HCT116 MMR-proficient cells treated with 6-MP (30 μM or 50 μM), and pretreated with the ROS inhibitor NAC (10mM). Results are expressed as percentage of cell viability and represent the mean ± SEM of three independent experiments. NAC cotreatment significantly hampered apoptosis induction by 6-MP (*P* ≤ 0.01). **D.** Quantitation of cell death using flow cytometry in HCT116 MMR-proficient cells treated with AZA (50μM or 100μM), and pretreated with the ROS inhibitor NAC (10mM). Results are expressed as percentage of cell survival and represent the mean ± SEM of three independent experiments. NAC cotreatment significantly hampered apoptosis induction by AZA (*P* ≤ 0.001). **E.** Western blot analysis of PARP level in response to ROS inhibition by NAC in cells treated as in C. Histogram shows the quantitation of PARP relatively to β-actin. **F.** Western blot analysis of PARP cleavage level in response to ROS inhibition by NAC in cells treated as in D. Histogram shows the quantitation of PARP cleavage relatively to β-actin. ROS scavenging by NAC leads to a marked increase in PARP level relative to a decrease in apoptosis in all 6-TG, 6-MP and AZA treated cells when cotreated with NAC. **G.** Western blot analysis of LC3 expression in response to ROS inhibition by NAC in cells treated as in B. Histogram shows the quantitation of LC3-II relatively to β-actin. **H.** Western blot analysis of LC3 expression in response to ROS inhibition by NAC in cells treated as in C. Histogram shows the quantitation of LC3-II relatively to β-actin. **I.** Western blot analysis of LC3 expression in response to ROS inhibition by NAC in cells treated as in D. Histogram shows the quantitation of LC3-II relatively to β-actin. ROS scavenging leads to a marked decrease in LC3-II level relative to a decrease in autophagy in 6-TG, 6-MP or AZA treated cells when cotreated with NAC.

ROS have important roles in intracellular signal transduction and redox homeostasis [31]. However, excessive ROS accumulation can exert toxic effects and cause oxidative stress, damaging main cellular components such as DNA, lipids and proteins, causing apoptosis [32–34]. To investigate whether thiopurine-induced apoptosis and autophagy was related to changes in the intracellular redox environment, we examined intracellular ROS production in thiopurine-treated colorectal cancer cells using dihydrorhodamine 123 (DHR). 6-TG, 6-MP and AZA caused a significant increase of ROS production in HCT116 MMR-proficient cells and in HT29 cells (Figure 6A).

ROS play an important role in apoptosis induction, and it has been reported that ROS could also regulate autophagy in various cell models [35, 36]. Therefore, the role of ROS production in apoptosis and autophagy induction by thiopurines was further confirmed by ROS scavenging in HCT116 and HT29 MMR-proficient colon carcinoma cells using *N*-acetyl cysteine (NAC) cotreatment.

Cell death induction by 6-TG, 6-MP and AZA was significantly inhibited using NAC cotreatment in HCT116 cells (Figure 6B, 6C and 6D) and in HT29 cells (Supplementary Figure S4A, S4B and S4C). These data were confirmed by a significant decrease in PARP cleavage in cells when cotreated with 6-MP or AZA and NAC (Figure 6E and 6F). Moreover, the appearance of the autophagy hallmark LC3-II was reduced in HCT116 MMR-proficient cells when cotreated with 6-TG, 6-MP or AZA and NAC (Figure 6G, 6H and 6I).

**Figure 7:**
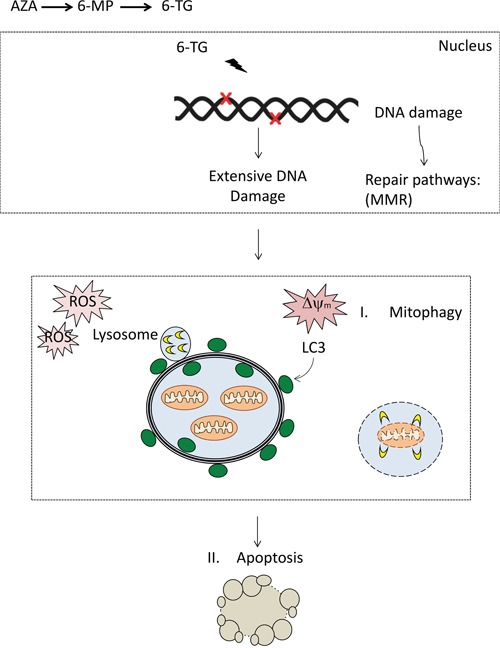
DNA damage by thiopurines accumulates, resulting in the activation of repair pathways including mismatch repair (MMR) The DNA damage signaling might transduce to mitochondria through P53. Expansion of the damage may result in the loss of mitochondrial membrane potential and ROS. Damaged mitochondria can be removed via autophagy and targeting to isolation membranes that then fuse with lysosomes, so-called mitophagy. Extensive ROS production and mitochondria damage may ensue apoptosis through opening of MTP and release of mitochondria intermembrane space proteins (MISPs) and the initiation of apoptosis. The apoptotic machinery lead to the activation of caspases and the execution of cell death.

These data provide strong evidence that ROS play an important role in thiopurine-induced cell death pathway, and maybe in the crosstalk between apoptosis and autophagy (Figure 7).

## DISCUSSION

Although various chemotherapeutics induce tumor cell death through the apoptotic machinery [37], apoptosis induction by chemotherapeutic agents cannot be considered as a default pathway, especially as defective apoptosis is considered a major hallmark of cancer cells [38]. It has been suggested that the ability of chemotherapeutics to kill cancer cells that display resistance to apoptosis may depend on the induction of autophagy and ultimately, autophagic cell death [39]. Autophagy induction has also been linked to resistance to chemotherapy through activating pro-survival pathways thus limiting the ability of the drug to kill cancer cells. The appropriate modification of autophagy, for instance, the inhibition of cytoprotective autophagy to enhance the apoptosis of tumor cells in response to anti-cancer agents might improve the effects of chemotherapy. Thus understanding how autophagy modulates cell fate in response to certain chemotherapeutics might improve their pharmacological effect.

Genetic variation in autophagy genes has been linked to susceptibility to Crohn's disease, [40, 41] which makes clarification of the involvement of autophagy in the thiopurine mechanism of action even more interesting and important. For instance, a recent study in two IBD cohorts have shown that a risk variant of the autophagy-related protein 16L1 (ATG16L1) gene associates with response to thiopurine treatment in patients with CD [42]. Risk allele carriers are proposed to have a better response in these studies. It could be hypothesized that Crohn's patients, carrying genetic variants in autophagy genes, might respond with a pronounced apoptosis rate, due to a lower rate of autophagy related processes, supported by the results in the present article.

We have investigated here the molecular mechanisms of thiopurine-triggered cell death. We found that autophagy is activated in response to 6-TG, 6-MP and AZA, in colorectal cancer cell lines proficient in MMR. Autophagy induction by the three thiopurines is a cell protective mechanism as its inhibition promotes cell death. On the other hand, apoptosis play a crucial role in cell death induction by 6-TG, 6-MP and AZA. Caspase inhibition using the broad range caspase inhibitor QVD protected the cells from cell death by thiopurines. More interestingly, apoptosis scavenging promotes the autophagic response to 6-TG, 6-MP and AZA, as a marked increase in the autophagic marker LC3-II was observed in the presence of QVD. Thus apoptosis and autophagy induction by thiopurines are interconnected. This suggests that a tight interplay between autophagy and apoptosis governs cell fate in response to thiopurines.

Earlier published data indicate that MMR processing of 6-TG initiates a prolonged cell cycle arrest followed by the activation of apoptosis and autophagy. Excess autophagy, resulting from continued MMR signaling, was suspected to lead to autophagic cell death [26]. Our data show that not only autophagy suppression leads to an increase in apoptosis induction by 6-TG, 6-MP and AZA. Surprisingly, inhibiting apoptosis did not lead to autophagic cell death, but rather protected the cells from cell death. These data demonstrate that apoptosis is the main cell death pathway by 6-TG, 6-MP and AZA and that autophagy activation by thiopurines is solely a prosurvival pathway in colorectal MMR proficient cells.

Links between autophagy and apoptosis are usually achieved via mitochondria. Induction of mitochondrial membrane permeabilization at a low level, below the threshold required for induction of apoptosis, results in sequestering damaged mitochondria in autophagic vacuoles. When mitochondrial membrane permeabilization is sufficiently high to sustain the active execution of cell death, apoptosis is induced [43]. Our data demonstrate that the mitochondrial pathway plays an important role in thiopurine-induced cell death as all three thiopurines; 6-TG, 6-MP and AZA, induce a loss of mitochondrial membrane potential, which is even greater when autophagy is inhibited. Autophagy plays a key role in clearing organelles that accumulate from the cytoplasm. This may become particularly important during oxidative stress in the presence of damaged mitochondria, a circumstance leading to increased accumulation of toxic ROS. We thus suspect that thiopurine-induced autophagy may play a role in the removal of damaged mitochondria as a marked increase in the loss of mitochondrial membrane potential is observed upon autophagy inhibition. Moreover, mitochondrial and autophagic signals colocalized in 6-TG treated cells, indicating the sequestration of damaged mitochondria in autophagic vacuoles during the activation of autophagy.

Aside to the decrease of Ψm observed upon 6-TG, 6-MP and AZA treatment, a marked increase of ROS production was also observed in MMR-proficient HCT116 cells and in HT29 cells in response to thiopurines. Interestingly, ROS scavenging using the anti-oxidant NAC inhibited apoptosis induction by 6-TG, 6-MP and AZA. Also, a marked decrease of the autophagic marker LC3-II was observed upon NAC cotreatment. Therefore, we conclude that thiopurines induce mitochondrial depolarization and an increase in ROS production. ROS production leads to the activation of mitophagy on one hand for the degradation of damaged mitochondria and to the activation of apoptosis on the other hand for the execution of cell death, when mitochondrial depolarization go beyond the threshold for the activation of the cell death machinery. ROS production was restricted to AZA treatment in immortalized human hepatic IHH cell line and in nontumor intestinal human colon epithelium HCEC cell line in a recent study [44]. The same study showed that ROS scavenging didn't affect the cytotoxicity of AZA, 6-MP and 6-TG in the indicated cell lines. While this difference might be linked to a difference in the treatment conditions, it's plausible that ROS production by 6-TG and 6-MP is restricted to tumor cells. Hence, it would be important to investigate ROS production along with the effect of ROS scavenging in a number of normal and tumor cell lines and to check whether thiopurines activate different pathways in tumor and nontumor cell models.

In conclusion, the present study shed new light on 6-TG, 6-MP and AZA-triggered cell death. We provide evidence that apoptosis induction by 6-TG, 6-MP and AZA is the main cell death mechanism involved in the cytotoxicity of thiopurines. Autophagy induction by thiopurines is solely a cell protective mechanism involved in degrading damaged mitochondria. Thiopurine-induced autophagy and apoptosis involves an increase of ROS production by mitochondria, subsequent mitochondrial damage and loss of mitochondrial transmembrane potential. Finally, we suggest ROS as the critical factor that links autophagy and apoptosis induction through mitochondria.

## MATERIAL AND METHODS

### Cells and cell culture

Human colorectal cancer (HCT116) cells are MMR-deficient (MLH1−, MMR−) because the *hMLH1* gene in these cells contains a base substitution that results in a termination signal at codon 252 (TCA→TAA) [45]. HCT116 cells with stable transfection of human MLH1 cDNA to restore MMR activity (MLH1+, MMR+) in the HCT116 cells as well as the empty vector control HCT116 cells (MLH1−, MMR−) were kindly provided by Dr Francoise Praz (Centre National de la Recherche Scientifique, Villejuif, France) [27]. HT29 cells, a human colorectal carcinoma cell line proficient in MMR, were kindly provided by Professor Stig Linder (Department of Medical and Health Sciences, Linköping University, Sweden). The cells were maintained in Dulbecco's modification of Eagle's Medium (DMEM, Cat. D6429, Sigma, St Louis, MO, USA) and supplemented with 10% FBS (Cat. 10270106 Gibco, Carlsbad, CA, USA) and 10,000 U/mL penicillin-streptomycin (Cat. 15140-122, Gibco).

### Drug treatment of cell lines

Cells were seeded at 20 000 cells per well per 6 well plate and allowed to attach and grow for 20 h prior to drug treatments. Based on our IC50 calculations (data not shown), cells were exposed to 3 μM or 6 μM 6-TG (Cat. A4882, Sigma), to 30 μM or 50 μM 6-MP (Cat. 852678, Sigma), or to 50 μM or 100 μM AZA (Cat. A3656, Sigma) for 24 hours, after which cells were incubated in drug-free medium for 72 additional hours.

### Measurement of apoptosis by flow cytometry

Apoptosis was quantified at the indicated time points after thiopurine treatment using Po-Pro staining (Cat. P3581, Molecular Probes, Eugene, OR, USA) and 7AAD staining (Cat. 559925, BD Pharmingen, San Diego, CA, USA) according to the manufacturer's instructions. Briefly, at the indicated time points, cells were harvested and washed twice with PBS, then stained in PBS with Po-Pro and 7AAD for 30 min on ice. Cells were analyzed using a Beckman Coulter Gallios flow cytometer (Beckman Coulter, Miami Lakes, FL, USA). Single positive cells for Po-Pro corresponded to the early apoptotic cells while double positive cells were considered the late apoptotic. Single positive cells for 7-AAD corresponded to the necrotic cells. The obtained data was analyzed using Kaluza 1.2 analysis software (Beckman Coulter).

### Measurement of mitochondrial membrane potential

Mitochondrial permeability transition was determined by staining the cells with tetramethylrhodamine methyl ester perchlorate (TMRM, Cat. T-668, Molecular probes). The cells were harvested and washed once with PBS then stained with TMRM 15 min at 37°C. Mitochondrial membrane potential was quantified by flow cytometric determination of the FL2 fluorescence of the cells. Data were collected and analyzed using Kaluza analysis software (Beckman coulter).

### Immunocytochemistry and confocal imaging

Cells were grown overnight on coverslips and treated as described in previous section. At the indicated time points, cells were washed with PBS and then fixed in 4% paraformaldehyde (Art. sc-281692, Santa cruz biotechnology, Dallas, TX, USA) for 20 min then permeabilized with 0.1% Triton X-100 (Cat. X100, Sigma-Aldrich, Saint Louis, MO, USA) and blocked in 0.1% BSA (Cat. A7906, Sigma-Aldrich, Saint Louis, MO, USA). To detect LC3, cells were incubated with an anti-LC3 antibody; (1:500) overnight then washed thrice with PBS. The LC3-antibody complexes were stained with the corresponding Rhodamine redX secondary antibody (1:200) then washed thrice with PBS. Mitochondria and lysosomes were stained with TMRM (250 nM) and LTR (1:2 500) respectively, in culture medium for 15 min prior to fixation with paraformaldehyde (4%).

The fluorescent images were then observed and analyzed with a Zeiss LSM 510 inverted laser-scanning confocal fluorescence microscope using a 60X objective (Carl Zeiss, Thornwood, NY, USA).

### Cell extracts and western blotting

The level of LC3-II and PARP-cleavage were detected by immunoblotting. Briefly, whole cell lysates were prepared from the treated cells at the indicated time points using 1X RIPA Buffer (Cat. 89901, Pierce, Thermo Fisher Scientific, Waltham, MA, USA) and according to the manufacturer's instructions. Proteins were separated by denaturing SDS-PAGE using the 4-15% Mini-Protean TGX gels (Cat. 4561083, Biorad, Hercules, CA, USA) or the 4-15% Mini-Protean TGX stain-free gels (Cat. 456-8083, Biorad) and then transferred onto PVDF membranes using the trans-blot Turbo Mini PVDF transfer Pacs (Cat. 170-4156, Biorad). The membranes were blocked in 5% non-fat dried milk in PBST supplemented with 0.1% tween20, (cat 1706531, Biorad) and then incubated overnight with the corresponding primary at 4°C overnight. The blots were then incubated with respective secondary antibody for 1 h after 3 times PBST washing. The membranes were further washed thrice with PBST prior to developing using Clarity Western ECL Blotting Substrate (Cat.1705060 Biorad). Images were collected and analyzed using the Image Lab software version 5.2.1 (Biorad).

### Antibodies and reagents

The following primary antibodies were used: mouse anti-β-actin (Cat. ab97037, Abcam, Cambridge, MA, USA), rabbit anti-LC3 (Sigma-Aldrich, L7543), rabbit anti-MLH1 (Cat. 554073, BD Pharmingen) and mouse anti-PARP (Cat. 9546 Cell signaling technology, Danvers, MA, USA). The following secondary antibodies were used; anti-Rhodamine RedX anti-rabbit antibody (Cat. R6394, Invitrogen, Grand Island, NY, USA). The broad range caspase inhibitor Q-VD-OPH (Cat. SML0063, Sigma) was added to the cells at a concentration of 25 μM immediately after treating the cells with thiopurines. CQ (Cat. C6628, Sigma) was added at 10 μM and 20 μM for 6 hours, 24 hours post thiopurine treatment. NAC (Cat. A7250, Sigma) was added at a concentration of 10 mM for 30 minutes prior to treatment with thiopurines.

### Statistical analysis

Data from flow cytometry (cell survival, ROS production and Loss of Ψ are presented as mean ± SEM (n=3). For comparison of one mean against a reference, standard single sample t-test was used and values represent single-sided p-values. For comparison between two treatments, two-sample independent t-test was used. For comparisons of means among more than two groups ANOVA with Dunnet's post hoc test was used. All statistical analyses were performed using the IBM, SPSS Statistics 23.0 (SPSS IN., IBM, Armonk, NY, USA) software with two-sided tests, with a p-value of ≤0.05 considered as statistically significant. Statistical significance are plotted as following: *P≤0.05, **P≤0.01 and ***P≤0.001.

## SUPPLEMENTARY FIGURES



## Abbreviations

6-TG: 6-Thioguanine
AZA: Azathioprine
6-MP: 6-Mercaptopurine
TPMT: Thiopurine methyltransferase
HGPRT: Hypoxanthine-guanine phosphoribosyltransferase
HPRT: Hypoxanthine-guanine phosphoribosyl transferase
MMR: Mismatch repair
CQ: Chloroquine
PCD: Programmed cell death
IMS: Inter-Membrane Space
TMRM: Tetramethylrhodamine, methyl ester
DSBs: Double-strand breaks
ROS: Reactive oxygen species
DHR: Dihydrorhodamine 123
NAC: n-acetyl cysteine
MAPK: Mitogen Activated Protein Kinase
