# Supplementary material for: Interconnections between apoptotic and autophagic pathways during thiopurine-induced toxicity in cancer cells: the role of reactive oxygen species
Source: Oncotarget. 2016 Sep 28;7(46):75616–34. doi: 10.18632/oncotarget.12313 (PMC5342765; doi:10.18632/oncotarget.12313)
Supplement: Supplementary file 1 [file oncotarget-07-75616-s001.pdf]

Interconnections between apoptotic and autophagic pathways during thiopurine-induced toxicity in cancer cells: the role of reactive oxygen species

SUPPLEMENTARY FIGURES

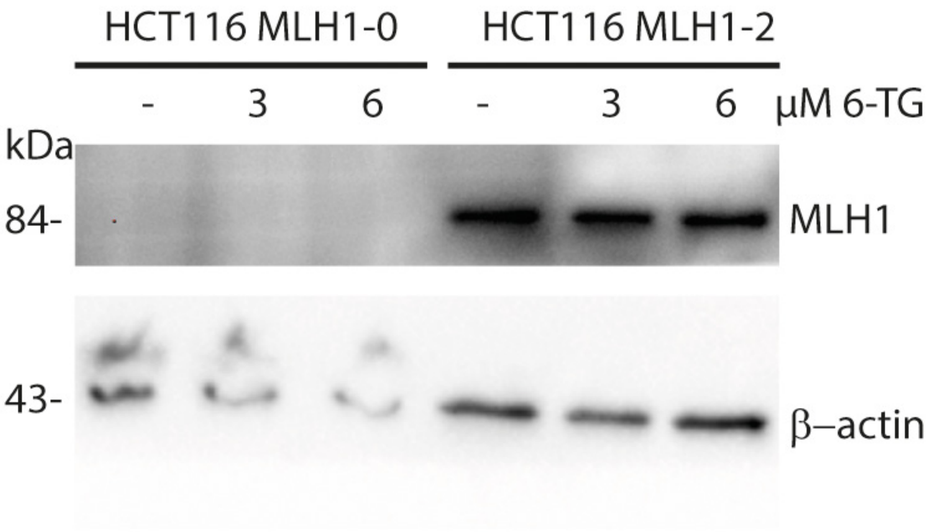

Supplementary Figure S1: MLH-1 expression in HCT116 MMR deficient (HCT116/MLH1-0) and MMR proficient (HCT116/MLH1-2) cells.

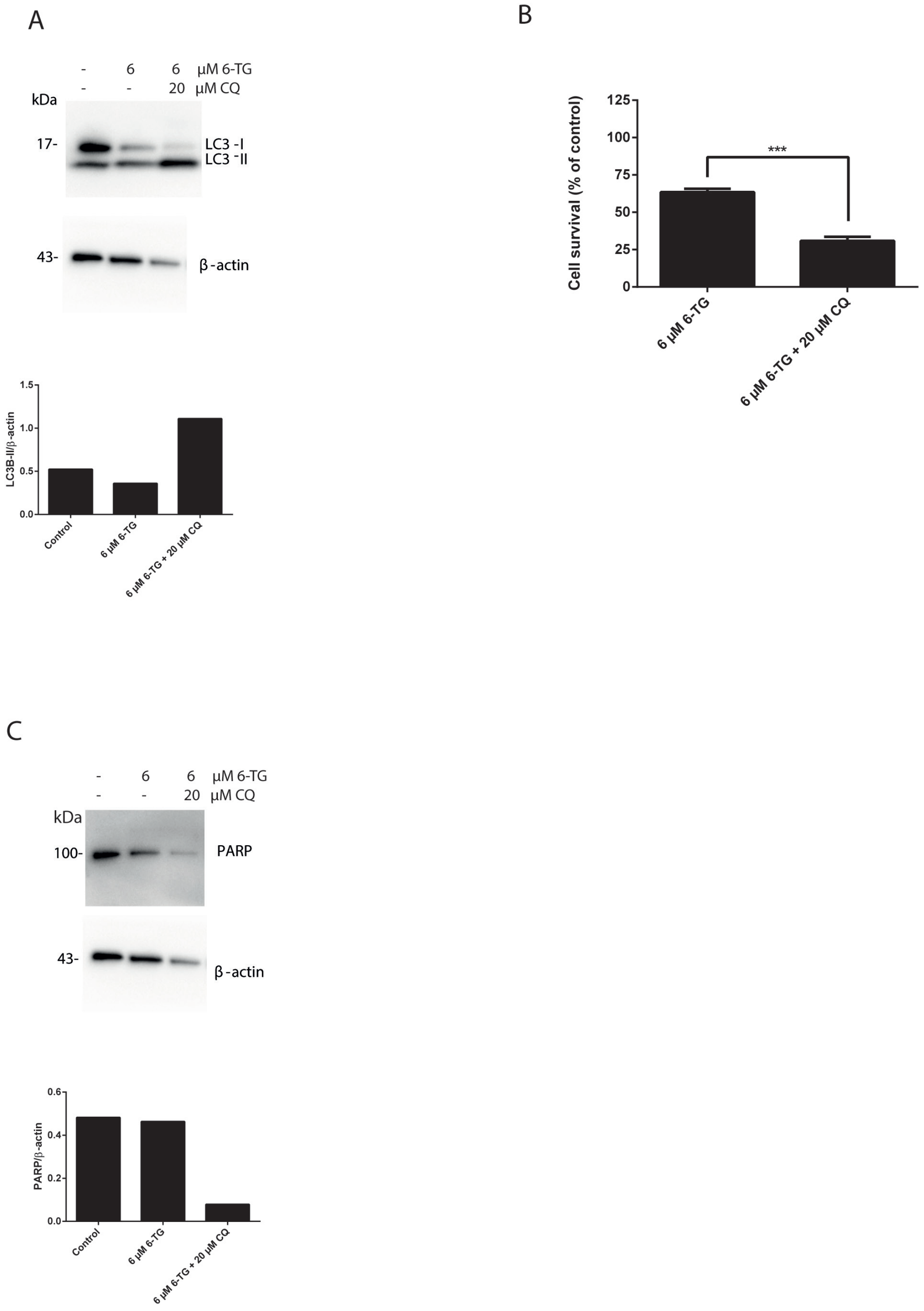

Supplementary Figure S2: A. Analysis of LC3 expression after 6-TG treatment of HT29 cells. Cells were treated with 6-TG (6 µM) for 24 hours followed by 6 hours treatment with CQ (20 µM) and analyzed by western blotting 72 hours later. Histogram shows the quantitation of LC3-II relatively to β-actin. CQ cotreatment leads to a marked increase in LC3-II level. **B.** Analysis of cell survival in HT29 cells. Cells were exposed to 6-TG for 24 hours followed by 6 hours treatment with CQ. Viability was determined 72 hours later using po-pro/7AAD staining and Flowcytometry. Results are expressed as percentage of cell survival and represent the mean ± SEM of three independent experiments. CQ cotreatment significantly increased apoptosis induction by 6-TG in HT29 cells ($P < 0.01$). **C.** Analysis of PARP levels in HT29 cells treated as in A. Histogram shows the quantitation of PARP relatively to β-actin. CQ cotreatment leads to a marked decrease in PARP level.

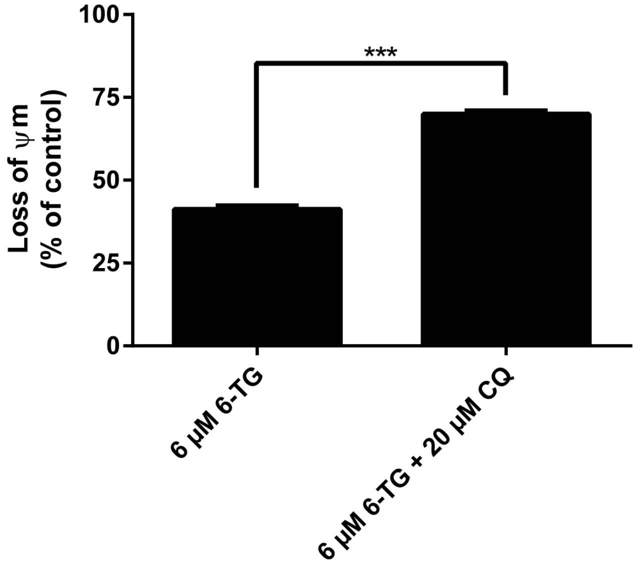

Supplementary Figure S3: Measurement of Ψm in HT29 cells treated with 6-TG (6 µM) and cotreated with CQ (20 µM). Results are expressed as percentage loss of Ψm and represent the mean ± SEM of three independent experiments. CQ cotreatment leads to a significant decrease in Ψm in HT29 cells (*P* < 0.001 and P<0.001 respectively).

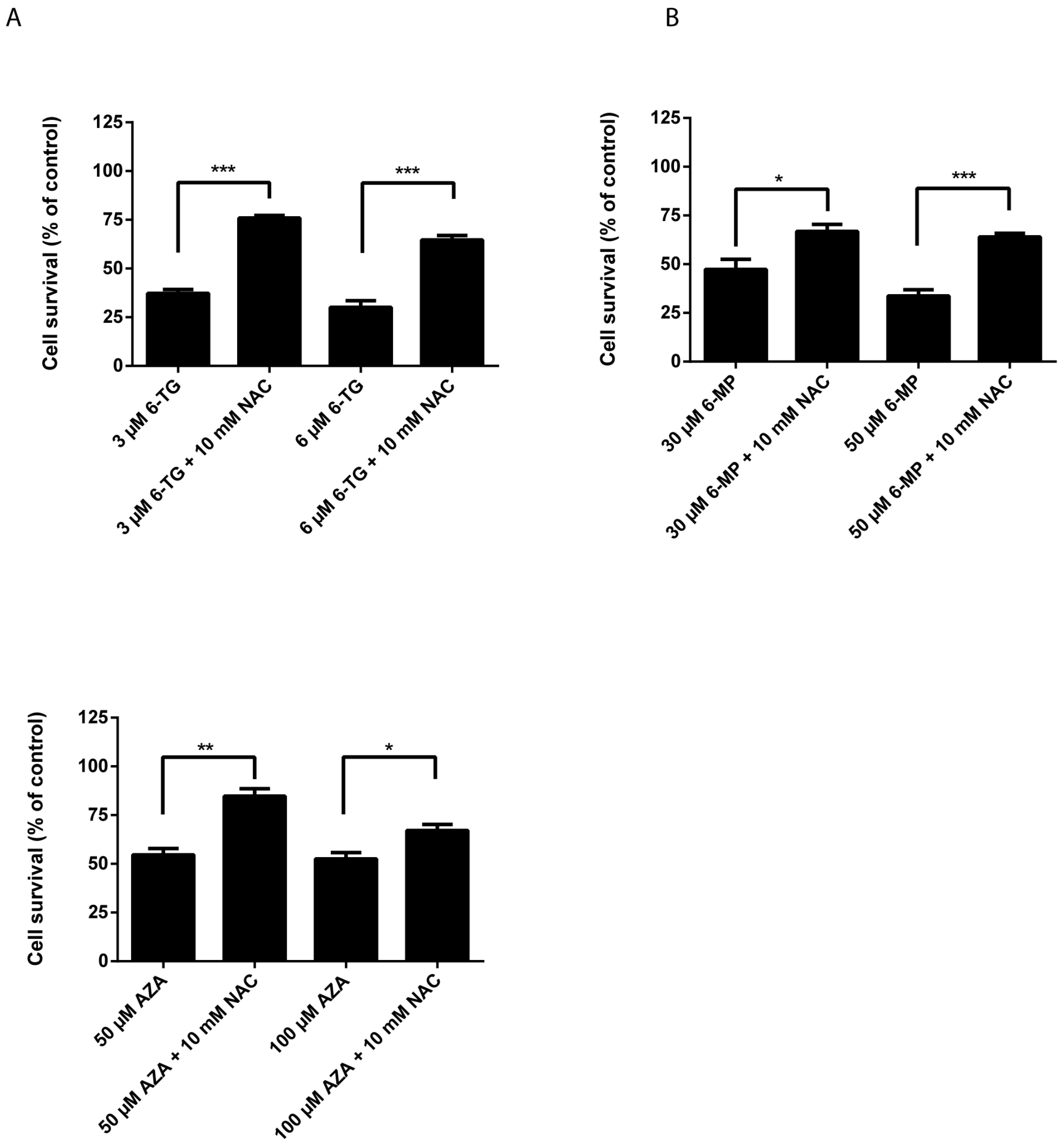

Supplementary Figure S4: A. Quantitation of cell survival using Flowcytometry in HT29 cells treated with 6-TG (3 µM or 6 µM) and pretreated with the ROS inhibitor NAC (10mM). Results are expressed as percentage of cell viability and represent the mean ± SEM of three independent experiments. NAC cotreatment significantly hampered apoptosis induction by 6-TG ($P < 0.001$). **B.** Quantitation of cell survival using Flowcytometry in HT29 cells treated with 6-MP (30 µM or 50 µM), and pretreated with the ROS inhibitor NAC (10mM). Results are expressed as percentage of cell viability and represent the mean ± SEM of three experiments. NAC cotreatment significantly hampered apoptosis induction by 6-MP ($P < 0.05$ and $P<0.001$ respectively). **C.** Quantitation of cell survival using Flowcytometry in HT29 cells treated with AZA (50µM or 100µM), and pretreated with the ROS inhibitor NAC (10mM). Results are expressed as percentage of cell viability and represent the mean ± SEM of three independent experiments. NAC cotreatment significantly hampered apoptosis induction by AZA ($P < 0.01$ and $P < 0.05$ respectively).
